# Characteristics and trends in acceptance and commitment therapy research: A bibliometric analysis

**DOI:** 10.3389/fpsyg.2022.980848

**Published:** 2022-11-14

**Authors:** Zhihong Li, Wenru Shang, Caiyun Wang, Kehu Yang, Juanmei Guo

**Affiliations:** ^1^Evidence-Based Medicine Center, School of Basic Medical Sciences, Lanzhou University, Lanzhou, Gansu, China; ^2^WHO Collaborating Center for Guideline Implementation and Knowledge Translation, Lanzhou, Gansu, China; ^3^The First Clinical Medical College of Lanzhou University, Lanzhou, China; ^4^Hospital Management Research Center, School of Management, Lanzhou University, Lanzhou, China

**Keywords:** acceptance and commitment therapy, knowledge map, research trends, depression, anxiety, bibliometric analysis

## Abstract

**Purpose:**

As acceptance and commitment therapy (ACT) becomes mainstream and a growing body of literature emerges, it is critical to map the global collaborative network and a quantitative and systematic assessment of ACT, as research on this topic is still lacking. This review aims to provide a comprehensive understanding of the trajectory, key themes, and future prospects in ACT research.

**Methods:**

Publications were extracted from the Web of Science Core Collection before 2022. Excel 2019, VOSviewer, and CiteSpace software were used to analyze the characteristics and trends of ACT research. Examples include publications trend analysis, authors’ cooperation network analysis, keywords co-occurrence analysis, and citation burst analysis.

**Results:**

A total of 799 articles in 314 journals contributed by 2,862 authors from 958 institutions in 52 countries were identified. The number of publications has increased significantly since 2015. The United States/Utah State University is the most productive country/institution; Karolinska Institute, Utah State University, and King’s College of London are the most significant nodes. Twohig M.P., Hayes S.C., and Levin M.E. are the most influential authors. Keyword co-occurrence analysis found the curative mechanisms, using network technology or mobile technology as adjuvant therapy, reducing psychological diseases of cancer patients were potential trends.

**Conclusion:**

This review is the first attempt of its kind to systematically examine the knowledge structure and draw an evidence map of ACT research. It deepens the understanding of existing research, gives many operable research directions and suggests to future ACT research.

## Introduction

Acceptance and commitment therapy (ACT) is a unique, experience-based psychological intervention, and is the third wave of behavioral therapy (Fernández et al., 2020; Ruiz et al., 2020). ACT was created in the late 20th and early 21st centuries by Steven C. Hayes, Kirk D. Strosahl, and Kelly G. Wilson, utilizing the relational frame theory (RFT) as its theoretical foundation and functional contextualism as its philosophical background. The RFT is a functional contextualist theory of human language and cognition that establishes connections through human non-organic formal mechanisms, and is generally characterized by “mutual derivability,” “joint derivability,” and “transformation of stimulus functions.” These components guide the development of ACT. Functional contextualism encompasses the understanding that psychological events are the continuous interaction between the whole and the specific situations (including history and environment). Functional contextualism also seeks to maintain the connection with the whole psychological event and its situation, analyzes psychological events in a way that maintains its integrity, aims to predict and affect the continuous interaction between the whole and the specific situations, and strives for the analysis to reach a certain accuracy, scope, and depth (Hayes, 2005; Hayes et al., 2012). In the past, patients often exhibited behaviors such as experiential avoidance, cognitive fusion, conceptualized past and fear of the future, attachment to the conceptualized self, lack of clear values, inaction, impulse or avoidance. Hayes illustrated that conflict between language/cognition and the environment is one of the root causes of dysphoria and that unwanted beliefs and suffering are associated with the response to mental illness, and that repression and avoidance not only do not free people from mental illness but lead to more pain. Subsequently, this then leads to creating, maintaining, and worsening psychological problems (Hayes et al., 2012). Compared with the previous treatment modes, the most important feature of ACT is to improve the psychological flexibility of patients, which can will enable patients to actively overcome these problems where they consciously make changes to be more aware and more fully connect with their own abilities in the moment, and actively and intentionally modify their behavior or make sustained efforts to achieve the established goals and values. Based on the ACT treatment mechanism, ACT emphasizes accepting unwanted experiences while encouraging value-driven behaviors to help individuals withstand unwanted emotions or change their understanding of and reactions to unwanted thinking and control of external actions to achieve their valued goals (Hayes et al., 2012; Houwer et al., 2016). Therefore, patients can contextualize the self with the help of ACT (Such as patients seeing themselves from an objective stance, observing everything, including their own perceptions, emotions, and will), clarify their values (through linguistically constructed aspirations and chosen direction of life), and commit to action (choosing behavior changes that are consistent with values, taking responsibility for actions, and supporting an effective values-based life; Houwer et al., 2016).

ACT has been therapeutic effects and utilized to address and manage symptoms including chronic diseases (Hayes et al., 1996; Wang, 2017), malignancies (Vowles and Mccracken, 2008; Xu and Yang, 2017), pregnancy and childbirth (Serfaty et al., 2016; Howard et al., 2022), psychiatric-psychological disorders (Lu and Fan, 2017; Waters et al., 2020), pain, depression, mixed anxiety, obsessive–compulsive disorder, psychosis, and so on (ACBS, 2022). This indicates that the ACT has important clinical significance, and they call for further follow-up and review of the development and effectiveness of ACT in future research is needed. Several recent studies have provided evidence-based literature reviews on ACT. For example, Graaf, et al. reviewed the existing literature on the treatment of chronic pain with online ACT. They found that most studies did not provide detailed information on the choice of optimizing the matching between tasks, technologies and users, and suggested that changes and key elements of the content, design and effectiveness of ACT should be considered in the future (Graaf et al., 2021). Osaji et al. reviewed and tested all the literatures about the ACT in treating various mental disorders and substance use disorder (SUD) management. They found that ACT has achieved great success in aiding the management of SUD, but more systematic and further research is needed in the future to analyze and track the overall progress of ACT (Osaji et al., 2020). Harris and Samuel analyzed the relevant literature on ACT interventions in adolescent mental health. They found that ACT contributes to addressing adolescent mental health problems; however, more insight is needed to the progress and changes of action to determine the role of ACT in increasing psychological flexibility (Harris and Samuel, 2020). Given this recent call to action, it is necessary to continue and expand ACT research. Bibliometric analysis is a method of quantitative analysis and visual analysis that analyzes existing literature on a specific topic. Compared with previous ACT studies, bibliometric analysis can objectively determine the overall layout of research topics, discipline advantages, research centers, and leading research teams from a multi-dimensional perspective, and determine the key areas of future research (Tjak et al., 2015; Li et al., 2020; Li Z. et al., 2021; Li, 2022). Bibliometric analysis is beneficial for document tracking and analyzing the knowledge structure of certain concepts. In the case of ACT, it may not only analyze the quantitative changes of ACT research but also summarize the concepts and research experiences of ACT, as well as highlight the areas that may need further research in the future. Bibliometric analysis can also combine author information, journal information, organization information, keyword information, geographic information and others to analyze the global research basis of ACT while highlighting the highlight the cooperation opportunities of ACT research in the future. Meanwhile, bibliometric analysis as a quantitative review method minimizes selection bias in the sampling process, which is open and visible, and provides higher-level evidence based on visual reviews of completeness. Thus, it reduces the dependence on experimental time and scope limitations (Tjak et al., 2015; Jin et al., 2021; Li, 2022; Zhang et al., 2022). Recently, bibliometric analysis has received increasing attention in various fields (Rixe et al., 2016; Kokol et al., 2020; Wg et al., 2021; You et al., 2021; Zhang et al., 2022). Therefore, it is necessary to provide an evidence-based, systematic, quantitative, transparent, and visual overview of ACT research, and this review attempts to answer the following questions.

Q1: What are the publication trends, the top journals, articles, authors, countries and institutions that contribute to ACT research?

Q2: What are the knowledge clusters in the knowledge structure of ACT research?

Q3: What are the future opportunities for ACT research?

Based on the above, this paper is structured as follows: first, we count the number of current ACT research works, including publication trends, the top journals, articles, authors, countries and institutions (Q1); second, we visually map the keywords to gain insight into the theoretical structure and current status of ACT research (Q2); third, we construct a co-citation analysis of authors, journals, and references to visualize the network relationships among them(Q2); and fourth, a burst analysis of connections is used to explore dynamic changes and new knowledge turning points in ACT research (Q3).

### Theoretical background

#### Philosophical background: Functional contextualism

ACT is developed from behavioral analysis. The core of behaviorism is environmentalism, which focuses on the changing and shaping of people that is caused by changes in the external environment. The philosophical basis of this phenomenon is contextualism (Hayes, 2005). Contextualism refers to understanding a psychological event as a continuous interaction between the whole and a specific situation (including history and environment), seeking to keep in touch with the whole psychological event and its situation, analyzing the psychological event in a way that does not destroy its integrity, and trying to predict and affect the continuous interaction between the whole and the specific situation, so as to allow the analysis to reach a certain accuracy, breadth and depth. Compared with the form of psychological events, contextualism pays more attention to the function of psychological events. As the philosophical background of ACT, functional contextualism is mainly embodied in several aspects. First, ACT operates from the belief that there is no absolute universal truth in the world and emphasizes the importance of effectiveness, considering personal values as the premise to measure effectiveness. Second, ACT’s behavioral analysis captures “prediction and influence” as the overall goal, which requires multi-dimensional contextual variables to be included in the analysis. If the context is not changed, it is impossible to have an impact on psychological events. Only context variables can be directly manipulated (Hayes et al., 2012). ACT focuses on the background and function of psychological events and not just the form and intensity of cognition and emotion. In addition, the pragmatic orientation of functional contextualism places particular importance on the goal, which is also emphasized by ACT, and thus the individual actively invests in their life in line with their own values and aims to achieve these life goals, rather than pursuing vague theories. Therefore, functional contextualism can be regarded as the extension and contextual interpretation of Skinner’s radical behaviorism.

#### Theoretical basis: RFT

The RFT is a comprehensive functional context model for the basic research of human language and cognition, which guides the development of ACT. From this perspective, ACT integrates the basic research science of human language and cognitive attributes, which can be tested through many empirical studies and clinical empirical interventions. The RFT holds that human beings have produced language in the process of evolution. Language is everywhere, whether in real social life or the inner world. Understanding language and cognition is the key to understanding human behavior. The core of human language and advanced cognition is the ability to acquire and be controlled by context. It can artificially connect and combine events and change the function of specific events according to these relationships. The background of psychological events includes personal, physical, social, biological, and cultural characteristics. The interaction history between individuals and their living environment can affect their experience of psychological events in current situations. People’s learning of the relationship between language and cognition has three main characteristics: The first is mutual entry or directionality. The second is a combinational entry. The third is that this relationship can transform the function of a related stimulus. When these three characteristics determine and form a specific relationship, we call this relationship a “relational frame” (Hayes, 2005; Hayes et al., 2012). Generally speaking, the RFT is a set of context models with functional meaning. The model’s core is human language and cognitive system, which means that human language is the key to understanding human behavior. This is because human language and some advanced cognitive skills possessed by human beings need to be acquired through learning and are always controlled and influenced by the external environment.

#### Therapeutic mechanism of ACT

Psychotherapy often faces three issues: the universality of pain, cognitive fusion, and empirical avoidance. The universality of pain means that people can feel pain in any situation; that is, people cannot solve their own pain by simply avoiding the environment. Cognitive fusion means that because of the establishment of relationship networks, thoughts or languages can be confused with all things involved. Experiential avoidance means that people tend to avoid their own internal experiences, including unpleasant physical feelings, emotions, thoughts, memories, and behavioral tendencies. At the core of these three issues is how to improve mental flexibility, which is also an important criterion for measuring mental health. Therefore, ACT aims to improve mental flexibility. Individuals should fully understand their abilities in this way and use their abilities to make concerted efforts to change and achieve set goals (Hayes et al., 2006). In summary, the therapeutic mechanism of ACT is divided into six processes:

1. Acceptance: In ACT, acceptance is not just tolerance but a positive, rather than a judgmental acceptance, of the experience in the moment. The aim is to make room for painful feelings, impulses, and emotions and not to try and resist, control, or escape them. The individual should aim to observe them objectively.
2. Cognitive dissociation: This refers to the separation of the self from thoughts, images, and memories and objectively observing such thought activities as a vehicle. This allows an individual to perceive thought as a language and text independently rather than something with meaning that it represents, which is entirely out of the individual’s control.
3. Being present: ACT encourages participants to consciously pay attention to the environment and psychological activities in the moment, without making any evaluations, and fully accept them.
4. The self as context: Painful thoughts and feelings threaten the participant’s self, and this negative feeling is particularly significant when the self is the conceptualized object. Observing the self can help participants pay attention to their own real experiences and promote cognitive dissociation and acceptance. ACT usually uses mindfulness, metaphor, and experiential processes to help visitors achieve self-observation.
5. Values: The values in ACT refer to the overall yearning and chosen life direction of participants, which are constructed through language. Values cannot be separated from people’s behavior, and they consciously run through every purposeful action in life. Values-based action is constructive and not an attempt to escape painful feelings.
6. Committed action: ACT is not only a treatment strategy for acceptance, but it is also a treatment strategy for changing orientation. The purpose of ACT is to help participants choose behavior changes that conform to their own values, take responsibility for their own actions, and support an effective value-based life.

The six core processes of ACT can be divided into two parts. The first part is the process of mindfulness and acceptance: ACT aims to help participants reduce subjective control, subjective judgment, language dominance, empirical escape, and experience life in the present through unconditional acceptance, cognitive dissociation, and attention to the present. Connecting with the here and now and one’s values leads to more flexible behavior. The second part is the process of commitment and behavior change: ACT helps participants mobilize and gather energy, move toward goals, and lead a valuable and meaningful life by focusing on the present, observing themselves, clarifying values, and committing to action.

#### Theoretical background and importance of bibliometrics

Bibliometrics is a quantitative statistical analysis tool that can quantitatively reveal the development history, research focus, and future research direction of an academic field (Tran et al., 2018). It was first proposed by famous British information scientist Allen Pritchard in 1969, marking the official birth of bibliometrics. The field has gone through three stages; germination, development, and differentiation. In the germination period (1917–1933), literature researchers pioneered the method of literature statistics, boldly aimed to conduct quantitative analysis on the literature of anatomy and chemistry in some disciplines, and achieved certain results. These studies laid the foundation for the creation and later development of bibliometrics. In the development process (1934–1960), theoretical research and legal discovery were the focus. Bradford’s and Zipf’s laws were one of the three basic laws of bibliometrics, which were discovered in this period. In the mature and differentiated stage, that is, the period of comprehensive development and differentiation (since 1960), bibliometrics developed from a narrow theoretical study to a wide range of applied research and indicator research involving more fields and topics (Kokol et al., 2020; Rejeb et al., 2021a,b, 2022).

The core laws of bibliometrics include Bradford’s, Zipf’s, and Lotka’s laws which are elaborated on in the following section.

Bradford’s Law: This law describes the law of document distribution. The number of papers published by a specialty is used to determine the core journals of that specialty. This law is used to guide literature and information work and scientific evaluation.

Zipf’s Law: This law is used to calculate the frequency of words in documents. Through the word frequency analysis of the literature, we can determine the research “hotspots” and trends of the discipline or industry.

Lotka’s Law: This law describes the relationship between the number of authors and the number of papers written. This current study discusses the law of balanced distribution of scientific paper authors. In the macro scientific writing activities, a few authors have written a large number of articles, whereas most of them have fewer works. According to this law, “the number of outstanding scientists is only the square root of the number of scientists” (Li, 2022; Yu et al., 2022).

Compared with traditional reviews, bibliometric methods can identify the importance of an article relative to other articles in the field and show how different articles are gathered together in the network. Therefore, the links between publications are visible, and some patterns or trends hidden from meta-analysis or structural review can be found. Further advantages of bibliometric methods include the accuracy and objectivity introduced in the evaluation of scientific literature. Researchers’ biases usually do not exist like in other types of review studies. Examples of this bias include excessive attention to specific authors and frequent self-reference (Wallin, 2005). Finally, when applying bibliometric methods, documents with a larger sample size can be easily carried out in hundreds of articles. Working with the same number of documents in a traditional overview can be otherwise cumbersome.

#### Early research and existing reviews

The existing research on ACT can be roughly divided into therapeutic mechanisms (or therapeutic processes) research and efficacy research. In terms of treatment mechanisms, several empirical studies have been carried out to explore the relationship between the core elements of ACT and the specific change processes, especially the main elements or methods involved in the psychological flexibility model. For example, with cognitive separation, a study using the time series paradigm found that when subjects were asked to quickly repeat a negative word related to themselves, they would reduce the negative emotions and beliefs related to the word. This study supports the necessity of using repetition vocabulary skills in cognitive dissociation (Masuda et al., 2008); regarding acceptance, the researchers compared the reaction of panic disorder patients with three strategies of acceptance, neutral description, and emotional repression through a strictly controlled laboratory research paradigm. The results showed that the anxiety levels and avoidance behavior of the receiving group were significantly lower than those of the depression group and the control group (Graaf et al., 2021). In terms of values, researchers discussed the impact of social psychological interventions on the behavior of disadvantaged ethnic students in real life (Cohen et al., 2006). In addition, some researchers compared ACT with Cognitive Behavioral Therapy (CBT) and found that ACT can reduce the credibility of the literal meaning of negative thinking leading to faster improvement (Bond and Bunce, 2000). In terms of efficacy research, researchers have found that ACT can effectively treat psychological health problems such as depression, obsessive–compulsive disorder, drug abuse, chronic pain, eating disorders, and work stress (Hayes et al., 1996; Wang, 2017; Graaf et al., 2021; ACBS, 2022). For example, ACT is more effective than cognitive therapy in treating depression; the effect of workplace stress management is better than that of workplace behavior adjustment training. In the treatment of social anxiety, ACT is more effective than group cognitive therapy (ACBS, 2022).

While some traditional review studies have been found and they have reviewed the role of ACT in treating of chronic pain, SUD, and adolescent mental health, and the latest systematic narrative review to study the current situation of the use of acceptance and commitment therapy (ACT) in local areas (Iran; Akbari et al., 2022), no comprehensive and timely bibliometric review has been found in the literature, which is a significant research gap this paper is aiming to fulfill. Due to the significant growth of academic literature in this field, it is necessary to apply bibliometric methods to better understand the knowledge structure of this field.

## Materials and methods

We conducted a bibliometric analysis related to the ACT research, and the literature data came from the Web of Science Core Collection (WOSCC) database.

### Eligibility criteria

To maximize the availability of ACT studies, we developed the following inclusion criteria with the advice of systematic evaluation experts. First, articles published between 1980 and 2021 in peer-reviewed English-language journals that included ACT-related search terms such as “acceptance and commitment therapy,” “ACT,” or “commitment acceptance” in context. These articles were eligible for inclusion in the citation analysis. Second, letters, conference abstracts, editorial materials, news, conference presentations, book reviews, and those without full text were excluded.

### Search strategy

Retrieved articles were identified by using ACT-related search terms for the WOSCC database, one of the world’s most extensive multidisciplinary repositories of scholarly information, at the same time, evidence shows that when visual analysis is conducted, the web of science database can provide better knowledge map effect (Falagas et al., 2008; Martin et al., 2018; Luo et al., 2021). Two researchers independently searched the database at the Center for Evidence-Based Medicine using the following specific strategies (Tian et al., 2017): Topic: (“Acceptance and Commitment therap*” OR “ACT*” OR “Commitment and acceptance method*” OR “Commitment Acceptance therap*”), spanning the period 1980 to January 1, 2022. “Topic” was selected to access more comprehensive relevant publications as much as possible. “Article,” “Early Access,” and “Review” were our target publication types because they had better and more up-to-date full-text information than other types of documents. The language was limited to English, and all retrieved studies are determined to be published before 2022.

### Data extraction

Two authors independently retrieved data from the WOSCC database; studies were identified by checking titles, abstracts, keywords, and full text after duplicate articles were removed. Exported records were “plain text” and “full records and citation references.” Any discrepancies or disagreements during the extraction steps were discussed and/or resolved by the team. The extraction of the proposed results is not limited to the title, journal, author, number of citations, year of publication, author affiliation, author region, impact factor (IF), and so on.

### Analysis method

**Figure 1:**
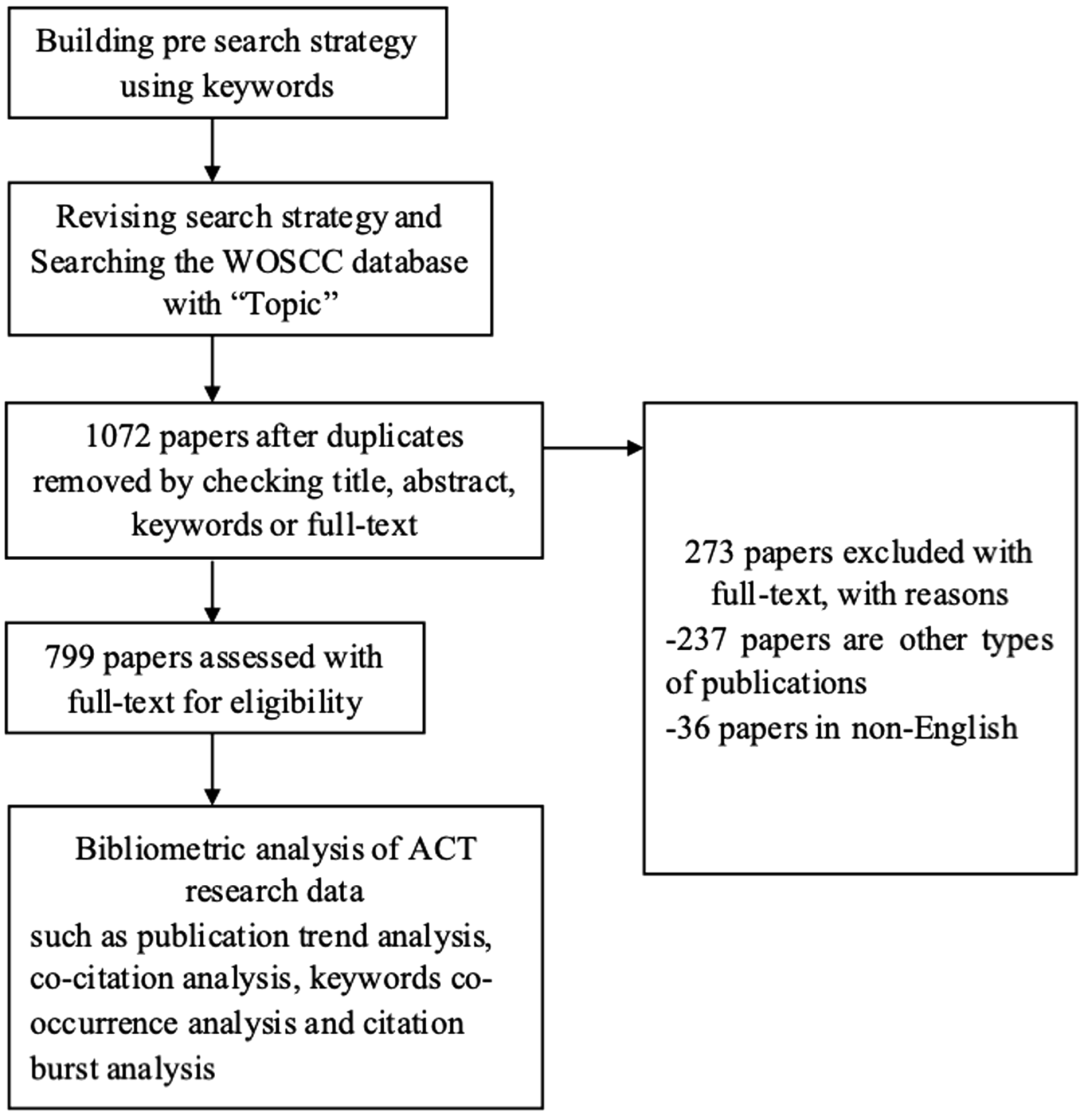
Data search and selection process in ACT research (based on the PRISMA 2020 statement).

The systematic bibliometric analysis follows the international standard process (Figure 1) and provides detailed information on each step (Page et al., 2021). The complete search strategy is shown in Appendix A. These included studies were conducted in Microsoft Excel 2019, CiteSpace, and VOSviewer. Microsoft Excel 2019 is mainly used for descriptive statistical analysis of collected article data, such as author, journal, publication year, author organization, fund source and IF, etc. VOSviewer is used to map authors, institutions, and keyword co-occurrence networks, whereas CiteSpace generates the most potent reference burst of keywords.

The data analysis resources were Excel 2020, VOSviewer (1.6.16), R (3.6.0) and CiteSpace (5.7.1.R5), and detailed parameter settings for the software are available *via* email. This computer uses a 64-bit macOS system (High Sierra 10.13.6) with a Java (1.8) environment.

## Results

Overall, the study covered 799 articles in 314 journals contributed by 2,862 authors between 1980 and 2021 (due to database inclusion restrictions, only publications from 2001 to 2021 were captured). These authors belong to 958 institutions in 52 countries. The total number of citations is 20,935, with an average of 26.2 citations per year.

### Annual trends in the number of publications

**Figure 2:**
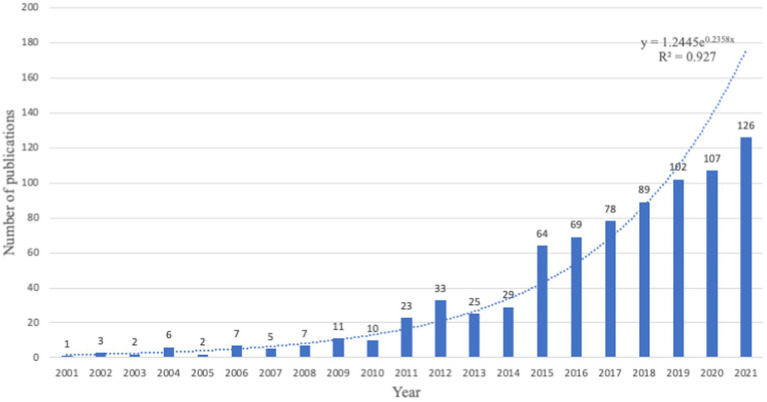
Annual output of ACT research.

Figure 2 shows the number and trend of publications published each year, with changes in the number of publications in a given discipline not only reflecting its current development but also predicting future trends. ACT as a mainstream therapy, although proposed in the 1990s, was only able to be examined from 2001 onwards in the web of science database due to search limitations, so 2001 became the starting point for this research. The changes can be divided into two stages: the first phase was from 2001 to 2004, the number of articles varied from 1 to 6 every year, and the number of publications increased slowly every year, the second phase was from 2005 to the present, first experiencing a stable period (2005–2008) with publications between 2 and 7 per year, and after 2008, the number of publications per year published more than ten and had significant growth trend, especially in the last 3 years (2019-present), the number of publications per year is above 100, and the growth is very substantial, which indicates that ACT has been progressing since its introduction as a mainstream therapy and is the fastest-growing in the last 3 years, and according to the fitted trend line *y* = 1.2445e^0.2358x^ (*R*^2^ = 0.927). Thus, the number of published articles in ACT research will maintain a double-digit growth trend.

### Journal distribution

A total of 799 articles were published in 314 different journals, of which 10 journals published more than 10 articles (3.2%), 106 journals published 2–9 papers (24.5%), and 198 journals published only one piece (72.3%). Table 1 lists the 10 most productive journals in ACT research, among which the “*Journal of Contextual Behavioral Science”* is the most productive journal, with 76 articles (9.5%) published and 569 citations, followed by “*Behavior Research and Therapy,*” which published 30 articles with the highest number of citations (4638), and then “*Behavior Therapy*” with 26 articles with 2,464 citations, and the last is “*Psychological Record,*” “*Trials*” and “*European Journal of Pain*” with eight or nine articles published and 54–251 citations. The 10 most productive journals’ research categories are mostly from psychology, general or internal medicine, and the neurosciences; the number of papers published in the top 10 journals is 0.5 times that of all other journals. This is an interesting phenomenon since most of these representative journals are interdisciplinary journals that belong to multiple discipline categories. For example, the “Journal of Contextual Behavioral Science” includes psychology and neuroscience, the “Frontiers in Psychology” includes psychology, management and medicine, and so on. Therefore, interdisciplinary integration will become the main research direction in ACT research.

**Table 1:** The top 10 most productive journals in ACT research.

Rank	Journal	Records	% of 799	IF-5 year	Total citations
1	Journal of Contextual Behavioral Science	76	9.512	3.828	569
2	Behaviour Research and Therapy	30	3.755	6.424	4,638
3	Behavior Therapy	26	3.254	5.425	2,464
4	Cognitive and Behavioral Practice	24	3.004	3.271	720
5	Behavior Modification	23	2.879	4.092	1,121
6	Clinical Case Studies	16	2.003	1.171	68
7	BMJ Open	12	1.502	3.424	36
8	Frontiers in Psychology	11	1.377	3.62	100
8	Journal of Consulting and Clinical Psychology	11	1.377	6.844	1,453
8	Journal of Pain	11	1.377	7.061	349
9	Psychological Record	9	1.126	1.603	251
9	Trials	9	1.126	2.606	54
10	European Journal of Pain	8	1.001	4.065	238

### Most productive countries/institutions

A total of 52 countries and 958 institutions have participated in ACT research, with the United States (336/799, 42.1%) contributing the most. Only one country published more than 100 articles (1.9%), 16 countries published between 10 and 97 articles (30.8%), 20 countries published between 2 and 9 articles (38.5%), and 15 countries published only 1 article (28.9%). In terms of region, European countries were the most productive with 410 papers (410/799, 51.3%), followed by North America (336/799, 42.05%), Asia (85/799, 10.6%), and Oceania (83/799, 10.4%). Table 2 shows the top 10 most productive countries and institutions, with the United States as the most productive country with 336 publications (42.1%), followed by the United Kingdom (97, 12.1%), and finally, Australia, Sweden, Iran, the Netherlands, Spain, Canada, Finland, Ireland, Germany, and China. These nations had between 18 and 83 publications (332, 41.6%). In terms of institutions, the vast majority were from the United States, with no institution publishing more than 100 ACT studies, the most productive being Utah State University in the United States with 64 ACT studies (64/799, 8.0%), followed by the Karolinska Institute of Sweden with 50 studies (50/799, 6.3%), another eight institutions published fewer articles with 16 to 38 publications, whereas among the others, a total of 658 institutions published only one article.

**Table 2:** The top 10 most productive countries/institutions in ACT research.

Countries/Regions	Records	% of 799	Rank	Institutions	Records	% of 799
United States (North America)	336	42.05	1	Utah State University (United States)	64	8.01
England (Europe)	97	12.14	2	Karolinska Institute (Sweden)	50	6.25
Australia (Oceania)	83	10.4	3	University of Nevada (United States)	44	5.5
Sweden (Europe)	78	9.76	4	King’s College London (England)	38	4.76
Iran (Asia)	66	8.26	5	Uppsala University (Sweden)	32	4.01
Netherlands (Europe)	32	4	6	Islamic Azad University (Iran)	22	2.75
Spain (Europe)	32	4	6	University of California San Diego (United States)	22	2.75
Canada (Europe)	28	3.5	7	University of Washington (United States)	20	2.5
Finland (Europe)	20	2.5	8	University of Queensland (Australia)	19	2.38
Ireland (Europe)	20	2.5	9	Karolinska University Hospital (Sweden)	18	2.25
Germany (Europe)	19	2.38	9	University of Jyvaskyla (Finland)	18	2.25
China (Asia)	19	2.38	9	University of Twente (Netherlands)	18	2.25
Scotland (Europe)	18	2.25	10	Fred Hutchinson Cancer Research Center (United States)	16	2

**Figure 3:**
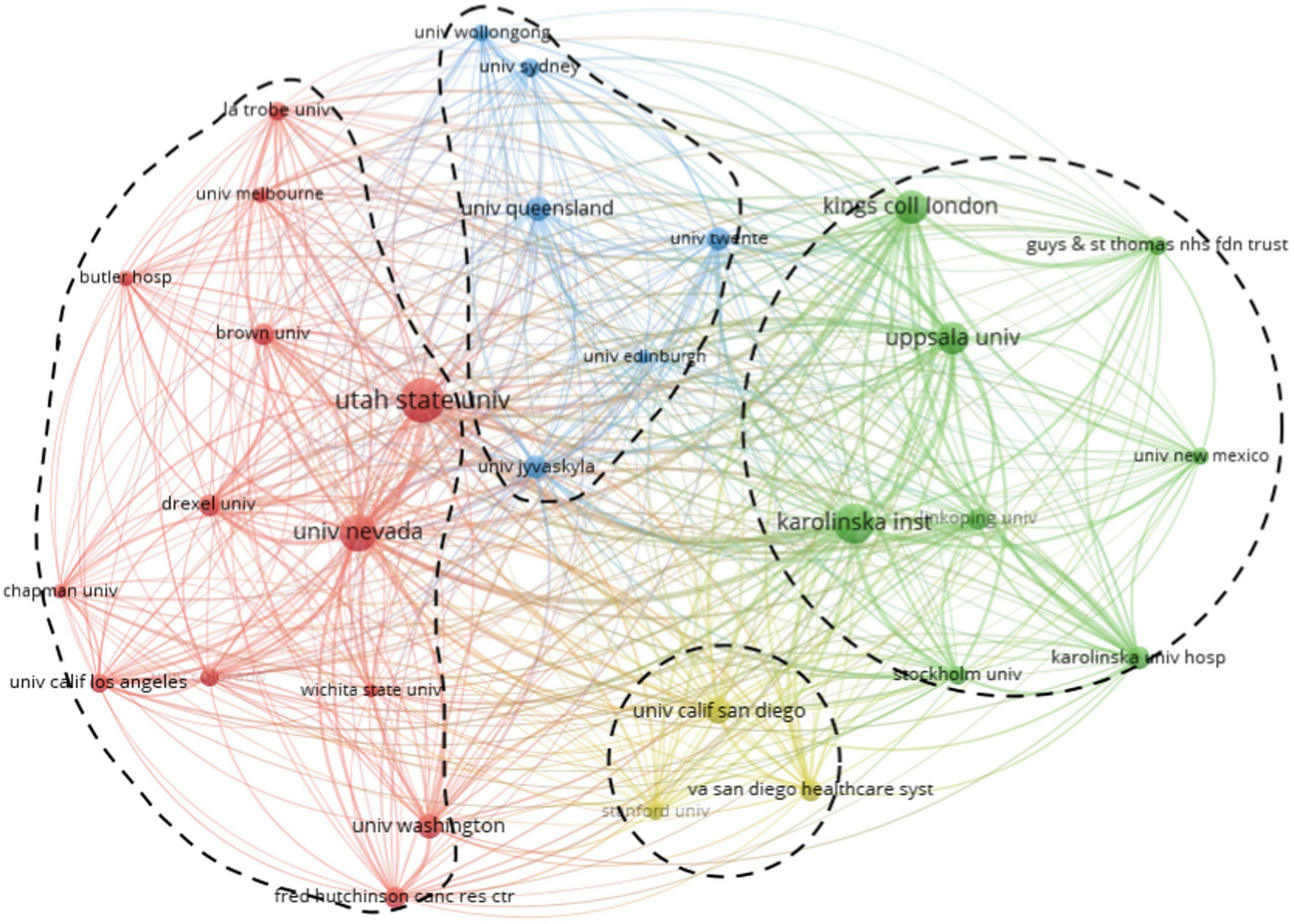
The network map of institutions from countries.

To obtain a better cooperation diagram, we selected the institutions that appeared nine times and finally got the top 30 most productive institutions, forming three main clusters. In general, there is close cooperation among these institutions, especially between institutions from the United States and the United Kingdom (Figure 3). The University of Nevada (United States) was the first representative to start ACT research. Later, other top institutions from the United States, Sweden, and the United Kingdom quickly followed up. Finally, they formed the most productive institutions at Karolinska Institute in Sweden, Utah State University (United States), and King’s College London (United Kingdom). However, no institutions were found in developing countries such as China. Therefore, future research on this topic requires active cooperation worldwide.

### Most productive authors and their cooperation

**Figure 4:**
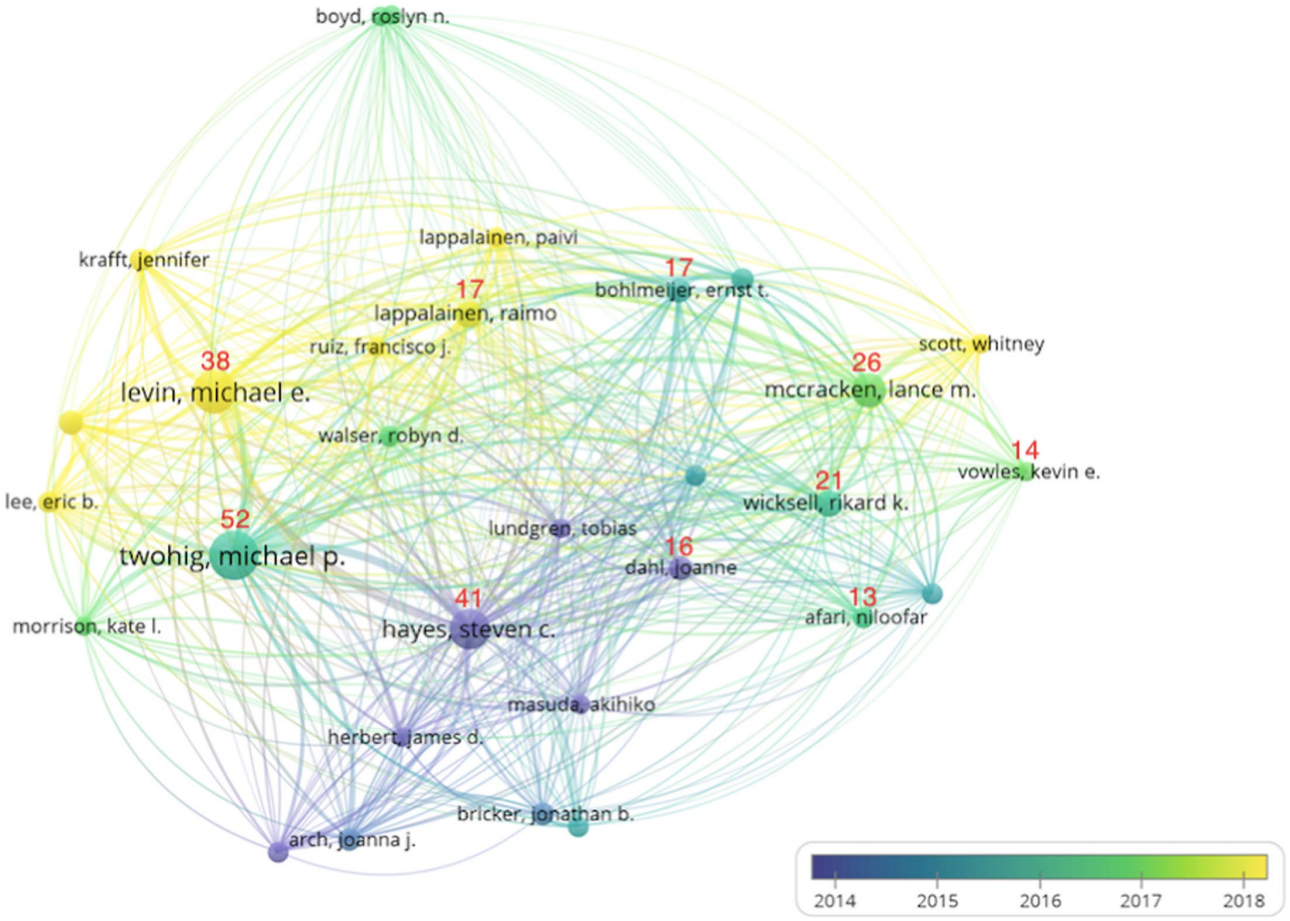
The overlay map of authors (top 30).

A total of 2,862 authors contributed to ACT research. We selected authors who contributed a minimum of eight articles to complete our author network graph. Finally, the top 30 authors met the criteria and formed an overlay visual collaboration graph with four main clusters (Figure 4) that are shown as darker bubbles representing the earliest authors, each node represents an author, the larger the node is, the more articles the author published, on the contrary, the fewer the nodes are. The lines between nodes represent the strength of the cooperation between authors, the thicker the lines are, the closer the cooperation between them is. The latter conducted ACT research, represented by thicker and more lines to represent closer collaboration. The number above the bubble represents the top 10 most productive authors and the number of articles contributed. Among the top 30 authors, the vast majority are from the United States. The first cluster (purple) is the team that first conducted ACT research, mainly in 2014, and is most represented by Hayes S.C. from the University of Nevada (41 articles, ranked 2) and Dahl J. (16 articles, ranked 8). The second cluster (green) is mainly focused on ACT research starting in 2015 and is also the most productive team, most represented by Twohig M.P. from Utah State University, United States (52 articles published, the most contributing author), Wicksell R.K. (21, rank 5), Bohlmeijer E.T. (17, ranked 6) and Afari N. (13, ranked 10). The third cluster (light green) is mainly focused on 2017. It is most represented by Mccracken L.M. (26, ranked 4) from Uppsala University, Sweden, and Vowles K.E. (14, ranked 9). The fourth cluster (yellow) is concentrated in 2018. The most represented are Levin M.E. (38, ranked 3) from Utah State University, the United States, and Lappalainen R. (17, ranked 7).

### Keywords analysis

A keyword analysis is a specific association between words based on the number of keyword co-occurrences, each node represents a keyword, and the distance between keywords is determined by the density between each node. The density represents the relationship and interaction strength between the two nodes. The goal of density-based algorithm is to minimize the weighted sum of the square Euclidean distance between each pair of nodes. Therefore, the higher the density value is, the closer the distance between the two nodes is, on the contrary, the farther it is (Rejeb et al., 2022). It is used to determine the relationships between topics in the disciplines represented in this literature set, which in turn, can assist in identifying areas (clusters) for current and future research that constitute the theoretical constructs or underlying themes of the research area (Eck and Waltman, 2010; Agarwal et al., 2016).

To ensure the accuracy and completeness of the keyword classification of the articles, “All Keywords” was chosen, which includes the keywords provided by the authors (Author Keywords) and the supplementary keywords provided by the web of science (Keyword Plus), which reduces the bias and risk of researchers limiting the content associated with manual tagging and does not quickly leave out potentially critical information provided in articles, and is effective when investigating the structure of knowledge in current and future scientific fields. In the implementation, we set the keywords to occur at least 13 times, and in the end, 100 words out of 2,380 keywords constituted the most vital set of available associated terms. The ten most important keywords were: acceptance and commitment therapy (527 records), depression (210 records), mindfulness (183 records), anxiety (165 records), cognitive behavioral therapy (146 records), chronic pain (132 records), psychological flexibility (117 records), psychometric properties (106 records), meta-analysis (100 records), randomized controlled trial (100 records).

**Figure 5:**
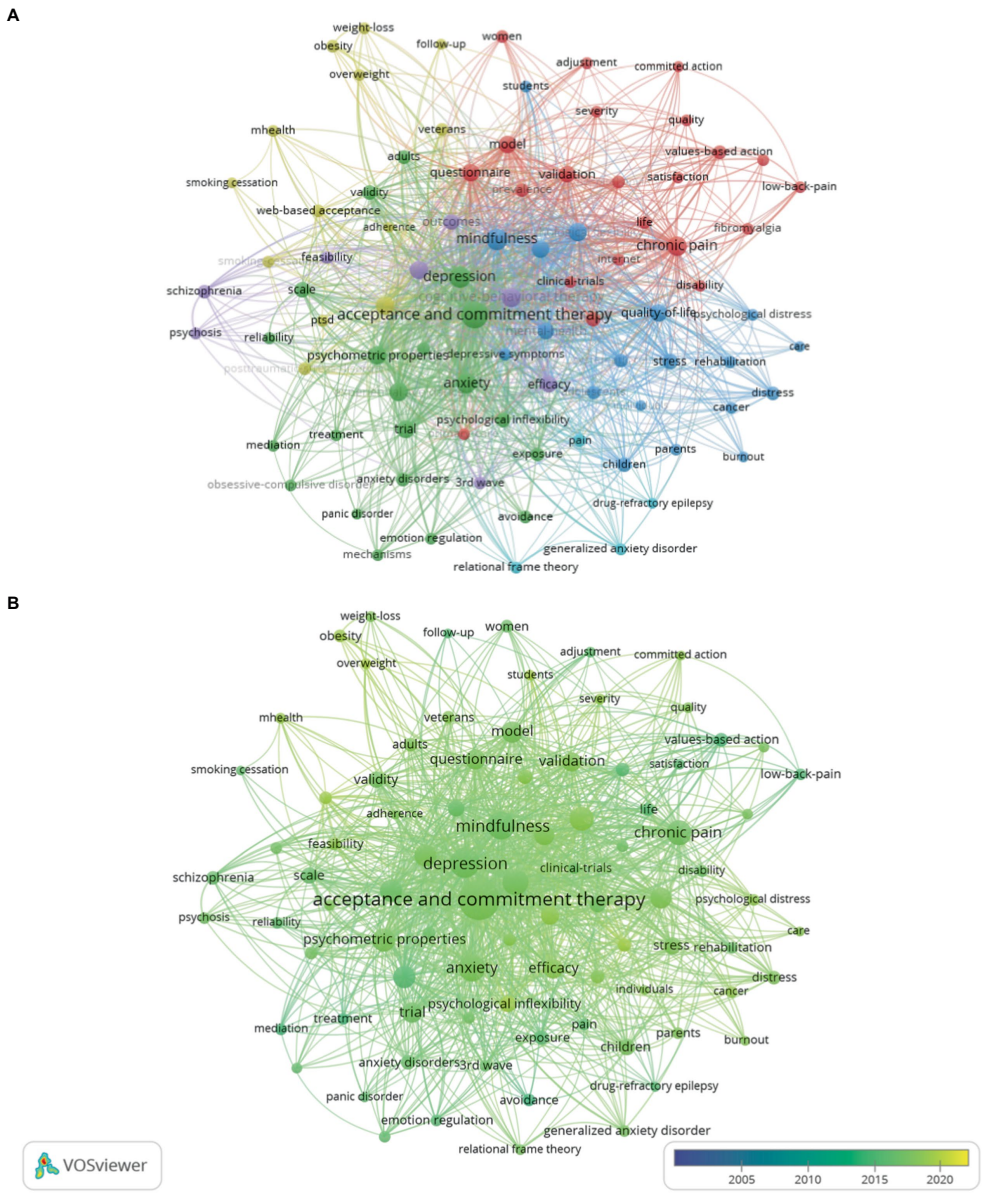
**(A)** The network map of keywords (top 100). **(B)** The overlay map of keywords (top 100).

From Figure 5A, six clusters are formed, the detail is shown in Supplementary material (Appendix 1).

Cluster-1 (green) focuses on the mechanism of ACT, including 34 keywords, this cluster is by far the most influential, because it includes the theoretical core (“psychological flexibility”), measurement (“scale”), mechanism (such as “mechanisms,” “mediation,” “validity,” “reliability”). The articles formed by these keywords constitute the basic background for understanding the concept, mechanism and relationship between ACT and other variables. It is likely to become a research point in the future to study the action mechanism of ACT on mental diseases through the action mechanism of mental illnesses (such as depression, anxiety, and panic disorder; such as psychological intelligence, emotional regulation, and psychological properties).

Cluster-2 (blue) focuses on the impact of the ACT on the psychological state (e.g., psychological distress, psychological flexibility) of cancer patients (e.g., children, parents). With the inclusion of 15 keywords, acceptance and commitment therapy has played an essential role in alleviating the mental health problems of cancer patients.

Cluster-3 (red) focuses on some ACT methods (such as models, questionnaires, and the Internet) as the primary nursing method to prevent pain (such as chronic pain, low back pain, or fibromyalgia) of patients (such as those with a disability or women), including 15 keywords.

Cluster-4 (purple) focuses on a meta-analysis to compare the effectiveness of CBT and ACT on the outcomes of schizophrenia. This included 19 keywords.

Cluster-5 (light blue) focuses on studying the mechanisms of pain, anxiety, and drug epilepsy by the ACT theory and includes 4 keywords.

Cluster-6 (yellow) compares on comparing the effects of network technology or mobile technology (such as mHealth, and web-based acceptance) on smoking, weight, PTSD, and weight loss by using an RCT test. This included 13 keywords.

In Figure 5B, keywords are determined by their frequency of occurrence and are given different colors depending on when they appear, with darker colors indicating earlier appearances in publications and lighter colors indicating more recent appearances in publications; the colors range from blue (earlier) to yellow (in recent years). The ACT research field has developed from the previous treatment of psychological diseases to the research on the influence mechanism of various patients’ psychological disorders, the use of some new network or mobile technologies for adjuvant treatment, and the breakthrough use of ACT to provide psychological care for patients with advanced cancer.

### Top cited articles and citation burst analysis of references

Table 3 shows the top 10 cited articles. These top-cited articles made outstanding contributions to ACT research, most of them published between 2002 and 2009, and three articles of them were from Hayes S.C., his article (Acceptance and commitment therapy: Model, processes, and outcomes) published in 2006 received the highest citation, with a total of 2,516 citations, ranking first and far ahead of other articles’ citations, which systematically described the treatment model and work on the ACT, while other articles published in the same period focused on the use of randomized controlled trials or meta-analysis to explore the treatment effects of ACT.

**Table 3:** The top 10 cited articles in ACT research.

Rank	Title	Authors	Journal	Year	Cited Times
1	Acceptance and commitment therapy: Model, processes and outcomes	Hayes SC, Luoma JB, Bond FW, et al.	Behaviour Research and Therapy	2006	2,516
2	Acceptance and commitment therapy, relational frame theory, and the third wave of behavioral and cognitive therapies	Hayes SC	Behavior Therapy	2004	833
3	The use of acceptance and commitment therapy to prevent the rehospitalization of psychotic patients: A randomized controlled trial	Bach P, Hayes SC	Journal of Consulting and Clinical Psychology	2002	415
4	A meta-analysis of the efficacy of acceptance and commitment therapy for clinically relevant mental and physical health problems	A-Tjak JL, Davis ML, Morina N, et al.	Psychotherapy and Psychosomatics	2015	339
5	A randomized controlled effectiveness trial of acceptance and commitment therapy and cognitive therapy for anxiety and depression	Forman EM, Herbert JD, Moitra E, et al.	Behavior Modification	2007	331
6	Acceptance and commitment therapy: A meta-analytic review	Powers MB, Vording VS, Emmelkamp PG	Psychotherapy and Psychosomatics	2009	325
7	Acceptance and commitment therapy and contextual behavioral science: Examining the progress of a distinctive model of behavioral and cognitive therapy	Hayes SC, Levin ME, Plumb-Vilardaga J, et al.	Behavior Therapy	2013	299
8	Acceptance and commitment therapy and mindfulness for chronic pain model, process, and progress	McCracken LM, Vowles KE	American Psychologist	2014	295
9	A randomized, controlled trial of acceptance and commitment therapy and cognitive-behavioral therapy for chronic pain	Wetherell JL, Afari N, Rutledge T, et al.	Pain	2011	256
10	Acceptance and commitment therapy and the treatment of persons at risk for long-term disability resulting from stress and pain symptoms: A preliminary randomized trial	Dahl J, Wilson KG, Nilsson A	Behavior Therapy	2004	238

**Figure 6:**
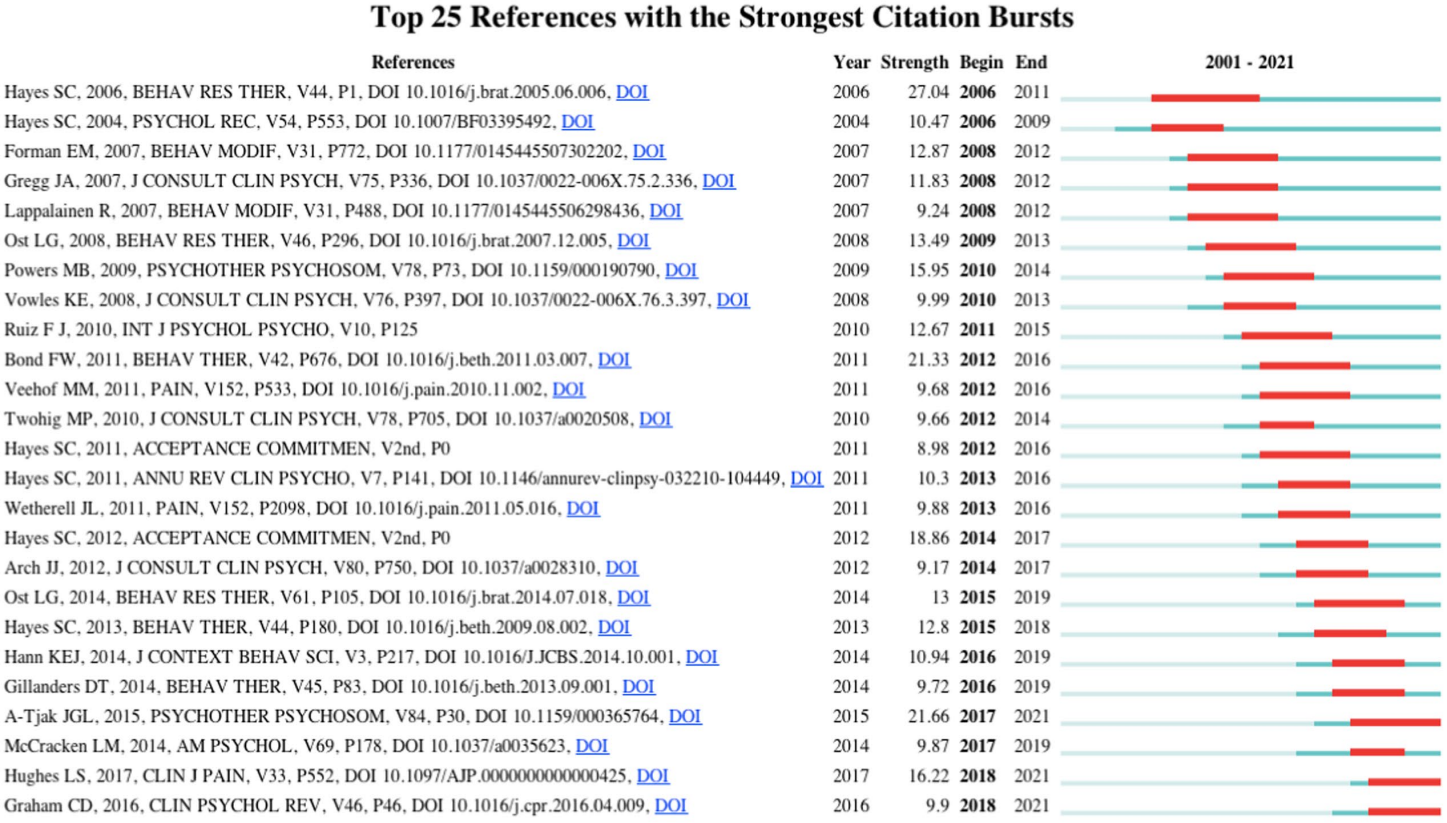
The top 25 references with strongest citation bursts in ACT research.

Figure 6 shows the citation burst analysis of 25 references. Citation burst analysis identifies and tracks publications with strong citation growth characteristics. This surge implies that an article has been cited rapidly over the past few years, marking a dynamic change in the research field and a new turning point in knowledge. Among them, Hayes SC’s “Acceptance and commitment therapy: Model, processes and outcomes,” published in 2006, has the highest intensity (27.04). Notably, Tjak et al. (2015), “A meta-analysis of the efficacy of acceptance and commitment therapy for clinically relevant mental and physical health problems” (cited 339 times, ranked fourth), Hughes et al., 2017, “Acceptance and commitment therapy (ACT) for chronic pain: A systematic review and meta-analyses” (152 citations), Graham et al. (2016), “A systematic review of the use of acceptance and commitment therapy (ACT) in chronic disease and long-term conditions” (110 citations), the outbreak of these systematic evaluation articles continues to this day, suggesting that systematic evaluation is also likely to be an essential focus for the field in the future.

## Discussion

This study conducted a systematic bibliometric analysis in ACT research based on the WOSCC database. It covered 799 articles published in 314 journals by 2,862 authors from 52 countries and 958 institutions between 2001 and 2021, with a total of 20,935 citations and an average of 26.2 citations per year. We analyzed the global ACT journal distribution, author output and collaboration, keyword co-occurrence, and a references’ citation burst analysis by visualizing and summarizing the structure, development, and future trends of ACT research. This was done to help future researchers understand dynamic changes in ACT research, new knowledge turning points, and identify future research directions (Agarwal et al., 2016; Kim et al., 2021).

The number of publications of ACT research has gradually increased and the worldwide cooperation has gradually been strengthened. Especially in the last 3 years (2019 to now), papers have increased significantly, with more than 100 published every year. From 2004 to 2018, the number of documents has shown an upward trend, with an annual number of 10–100. From 2001 to 2004, it has shown slow growth, with a yearly number of 1–6. This shows that ACT as a mainstream therapy in the world has been widely concerned by researchers and gradually applied (Eck and Waltman, 2010; Li H. et al., 2021). However, through a survey of research directions, we found that most ACT studies came from the United States, and the research areas varied widely, such as 448 papers in psychology, 171 in psychiatry, and 71 in neurosciences neurology. In contrast, the other directions account for a tiny percentage. One of the reasons for this unbalanced state may be the different perspectives, experimental designs, and studies in different directions of ACT (Barida and Widyastuti, 2019; Li H. et al., 2021; Tan et al., 2021; Reyes, 2022). Therefore, it is crucial for the evolving field of ACT to periodically review and evaluate its evolutionary pathways, and we hope to motivate future researchers to enhance interdisciplinary and integrative research to promote and coordinate ACT research.

As for journal distribution, most of them were published in medical and psychological journals. Among them, the “*Journal of Contextual Behavioral Science*” is the most productive journal, with 76 articles (9.5%) and 569 citations, followed by “*Behavior Research and Therapy,*” with 30 articles and 4,638 citations. The third is “*Behavior Therapy,*” with 26 articles and 2,464 citations. Only 10 journals published more than 10 (3.2%) articles, while 198 journals published only one article (72.3%). Although ACT has received widespread attention and research in various fields, publication quality needs to be improved. Although many articles were published in psychology, psychiatry, neurosciences neurology, general internal medicine, etc., the percentage of papers published in the top journals in these fields was meager (less than 10%). ACT is prevalent in these fields, and more different areas are starting to pay attention, but the quality of research needs to be further improved. Therefore, future researchers need to strengthen the theoretical basis, experimental design, and data analysis.

Within the most productive countries and institutions, most ACT studies came from countries and institutions in Europe and North America, with the United States being the most productive country (336/799, 42.1%) and the only country with more than 100 published articles (1.9%). The most influential institution was Utah State University in the United States, with 64 published ACT studies (64/799, 8.0%). We found that the vast majority were from institutions in developed countries, with few from developing ones. Our quantitative analysis helps to illuminate the relative contributions of individual countries and institutions to ACT research. However, our analysis of country/institution collaboration revealed very few collaborative networking relationships between countries and institutions, particularly between developed and developing institutions, suggesting that ACT is unequally developed across countries/institutions (Glanzel and Moed, 2002; Balter et al., 2021; Galvez et al., 2021). Therefore, we encourage research collaboration across institutional and national boundaries to discover differences between ACT studies and thus promote ACT research.

Keyword co-occurrence analysis Can better track the research trend of ACT, provide directional guidance for future research (Glanzel and Moed, 2002; Li Z. et al., 2021; Li, 2022), and also contribute to the theoretical and application development of ACT. By analyzing the evolution of ACT keywords, we found that keywords (such as “acceptance and commitment to treatment,” “depression,” “anxiety,” and “chronic pain”) maintained a stable and growing trend. This shows that these topics still have great research potential and significance in future research. In addition, we have recently identified some new research topics. The first is to study the treatment mechanism of ACT, which is also one of the core research topics of functional contextualism. The core of ACT is to improve the psychological flexibility of patients. Future research needs to conduct more in-depth measurements of the six processes of implementation, such as keyword co-occurrence analyses. Innovative research on the treatment mechanism and action mechanisms (such as “psychological rigidity” and “model”) is called for, using more diversified perspectives and measurement models to measure each link of patients treated with ACT and to further study the treatment mechanisms and action mechanisms of ACT. Theoretical innovation is carried out based on ACT theory (such as “relational framework theory”). Although it is difficult to innovate the RFT, theoretical innovation is still a very encouraging and popular research field. Recent literature findings call for comprehensive measurement of individual and group psychological flexibility and differences, and description of their thoughts and feelings when engaging in specific activities or behaviors, exploratory measurement of their values changes, and better observation of patients’ behavior changes will be more meaningful and practical. The second is to use some new network or mobile technologies for adjuvant therapy. Keyword co-occurrence analysis found that innovative research methods (such as “randomized controlled trial” and “meta-analysis”) and new technological innovations (such as “mhealth” and “network-based”) are frequently mentioned keywords. With the development of big data technology and mobile network technology, it will be more convenient and accurate to observe patients themselves and measure patients’ psychological flexibility, value changes, and action changes. The third is the breakthrough use of ACT to provide psychological care for patients with advanced cancer and explore more diseases (such as “cancer,” “smoking,” “overweight,” “PTSD,” and “weight loss”). These frequently occurring keywords indicate that ACT has been explored and used in a wider range of diseases and has achieved positive effects. These will be new trends and potential trends in ACT research. Therefore, these valuable and promising topics may attract more researchers and performers and help us understand ACT research.

### Theoretical and practical contributions

Our review provides some theoretical contributions to the ACT research. First, we used systematic quantitative bibliometric techniques to explore a large number of ACT studies and identified several core themes, such as therapeutic mechanism research, auxiliary use of new technologies, exploration of new disease treatment fields, etc. The discovery of these themes will help deepen and provide new perspectives and evidence for understanding the philosophical basis of relational framework theory and functional semantics. Second, it is different from previous comments on ACT, this research adopts an objective, systematic and transparent analysis method to construct relevant research topics and reveal the future research approaches of ACT. Through the analysis of cited journals, cited authors, cited articles and their keywords in ACT related fields, researchers can quickly understand the evolution process and knowledge structure of this field and provide new ideas for conducting new research.

Our review provides some practical contributions to the ACT research. Due to the adverse effects of drug therapy and the high requirements of physical therapy, cognitive therapy has become a hot research topic in the treatment of depression in recent years. It not only helps patients recover their standard psychological coping mechanisms but also improves their social function, which is of positive significance in preventing repeated attacks. Therapists actively guide patients to consciously accept and pay attention to the present, allow them to feel and accept love from family, nurses, and society, form a correct outlook on life and values, and offer positive psychological hints to patients. Then, through some practical activities, they consolidate effective values, promote patients to have positive coping behaviors, improve treatment confidence, effectively cooperate with nurses to conduct appropriate treatment, and help patients actively integrate into life. This reduces the psychological pressure on patients. A hospital is an organization that directly provides people-oriented treatment, services, and nursing for patients. Improving the treatment effects and nursing quality of patients, as well as reducing the psychological burden of patients, are the core implementation methods of medical, hospital, and nursing managers. In addition, nursing managers must improve their awareness and training on ACT therapy. For medical staff and researchers, bibliometric analysis can help these professionals quickly find hot topics, potential application fields, and highly cited articles in this field, which will provide them with evidence-based references. At the same time, the results of a bibliometric analysis can help medical staff use some new technologies to improve the nursing quality of patients. For example, ACT, assisted by information technology can reduce the psychological burden of patients, which is also the core implementation method of medical, hospital, and nursing managers. It can not only help patients restore normal psychological coping mechanisms but also improve patients’ social function, which is of positive significance in preventing recurrent attacks. We recommend that researchers and nursing managers use it to study other different diseases or mental patients.

### Strengths and limitations

Although the bibliometric method is very effective, there are still some limitations to be recognized. Firstly, although the Web of Science is one of the most widely used databases in visualization research, it is not the source of the whole act research literature; Therefore, we search based on the title and are limited by the WOSCC database. We cannot rule out the possibility of missing some data, such as the literature before 2001. If visualization technology matures in the future, we encourage the collection of publications from as many different database sources as possible to improve our research (Yan et al., 2019; Li Z. et al., 2021). Second, the included ACT research focuses on medicine or psychology; however, this is a topic of global communication and may also exist in business, the environment, or other fields. Therefore, future research needs to expand the scope of research, which may help clarify the literature in other fields other than medicine or psychology. Third, although we describe the production situation of scholars, journals, institutions, and countries, as well as the hot and cutting-edge trend analysis in the bibliometric analysis that is based on keywords, which may make it may be difficult to obtain all key information and deeply discover the potential links of these relationships. Further work may increase their deeper cooperative relationships.

## Conclusion

In summary, this study may provide a set of evidence for researchers, practitioners, and decision-makers engaged in psychotherapy to use ACT. Since 2015, the number of publications has increased significantly. The United States and Utah State University is the most productive and cooperative country and institution, respectively. Twohig M.P., Hayes S.C., and Levin M.E. are the most influential authors. The most productive core journal is the *Journal of Contextual Behavioral Science*. Research hotspots on the mechanism of ACT and the use of network technology or mobile technology to assist in the treatment and reduce the psychological diseases of cancer patients are potential concerns of future research. The contribution of ACT research and authors’ cooperation is strong in developed countries; global cooperation needs to be strengthened with developing countries to expand and deepen the understanding of ACT research.

## Data availability statement

The original contributions presented in the study are included in the article/Supplementary material, further inquiries can be directed to the corresponding authors.

## Author contributions

ZL and WS are co-first authors and conducted the design, collection, analysis, and wrote the draft. ZL conceived the study. WS, CW, JG, and KY provided methodological guidance or grant support for the study. All authors contributed to the article and approved the submitted version.

## Funding

This work was supported by the Major projects of NSFC: Theoretical system, international experience and Chinese path of evidence-based Social Sciences (grant no. 19ZDA142).

## Conflict of interest

The authors declare that the research was conducted in the absence of any commercial or financial relationships that could be construed as a potential conflict of interest.

## Publisher’s note

All claims expressed in this article are solely those of the authors and do not necessarily represent those of their affiliated organizations, or those of the publisher, the editors and the reviewers. Any product that may be evaluated in this article, or claim that may be made by its manufacturer, is not guaranteed or endorsed by the publisher.

## Supplementary material

The Supplementary material for this article can be found online at: https://www.frontiersin.org/articles/10.3389/fpsyg.2022.980848/full#supplementary-material
