# Supplementary material for: Characteristics and trends in acceptance and commitment therapy research: A bibliometric analysis
Source: Front Psychol. 2022 Nov 14;13:980848. doi: 10.3389/fpsyg.2022.980848 (PMC9702511; doi:10.3389/fpsyg.2022.980848)
Supplement: Supplementary file 1 [file Table_1.DOCX]

**Appendix A**

Web of Science Search Strategy

#1 Topic: ACT*

#2 Topic: Commitment and acceptance method*

#3 Topic: Acceptance and Commitment therap*

#4 Topic: Commitment Acceptance therap*

#5 #1 OR #2 OR #3 OR #4

Search in: Web of Science Core Collection Editions: All

Platform/Interface: Thomson Reuters.

Timespan: 1980–2021.

Result: 1072
